# Formation mechanism of Ruddlesden-Popper-type antiphase boundaries during the kinetically limited growth of Sr rich SrTiO_3_ thin films

**DOI:** 10.1038/srep38296

**Published:** 2016-12-06

**Authors:** Chencheng Xu, Hongchu Du, Alexander J. H. van der Torren, Jan Aarts, Chun-Lin Jia, Regina Dittmann

**Affiliations:** 1 Peter Grünberg Institute (PGI-7), Forschungszentrum Jülich GmbH, 52425, Jülich, Germany; 2 Ernst Ruska-Centre (ER-C) for Microscopy and Spectroscopy with Electrons, Forschungszentrum Jülich GmbH, 52425, Jülich, Germany; 3 Central Facility for Electron Microscopy (GFE), RWTH Aachen University, 52074, Aachen, Germany; 4 Leiden University, Huygens Kamerlingh Onnes Lab, NL-2300 RA, Leiden, Netherlands; 5 Peter Grünberg Institut (PGI-5), Forschungszentrum Jülich GmbH, D-52425, Jülich, Germany

## Abstract

We elucidated the formation process for Ruddlesden-Popper-type defects during pulsed laser deposition of Sr rich SrTiO_3_ thin films by a combined analysis of *in-situ* atomic force microscopy, low energy electron diffraction and high resolution scanning transmission electron microscopy. At the early growth stage of 1.5 unit cells, the excess Sr results in the formation of SrO on the surface, resulting in a local termination change from TiO_2_ to SrO, thereby forming a Sr rich (2 × 2) surface reconstruction. With progressive SrTiO_3_ growth, islands with thermodynamically stable SrO rock-salt structure are formed, coexisting with TiO_2_ terminated islands. During the overgrowth of these thermodynamically stable islands, both lateral as well as vertical Ruddlesden-Popper-type anti-phase boundaries are formed, accommodating the Sr excess of the SrTiO_3_ film. We suggest the formation of thermodynamically stable SrO rock-salt structures as origin for the formation of Ruddlesden-Popper-type antiphase boundaries, which are as a result of kinetic limitations confined to certain regions on the surface.

The prototypical perovskite material SrTiO_3_ (STO) exhibits a broad variety of functional properties, which might be of interest for future electronic applications such as redox-based memristive devices^1^, as well as for energy application such as catalysis^2^ or thermoelectricity^3^. Although device materials typically require unparalleled levels of purity and perfection, the presence of point and extended defects in SrTiO_3_ have been identified to be advantageous for a variety of applications. Oxygen vacancies induced by acceptor doping improve the ion conductivity^4^ and are prerequisite for the operation of redox-based memristive devices^1^. Cation vacancies, which are common defects in STO thin films^5^, reduce the thermal conductivity^3^, the permittivity^6^ and deteriorate the electron mobility of n-doped STO^7^. However, it has been reported recently that the formation of Sr vacancy clusters can strongly enhance the electron mobility in Ti rich n-doped STO^8^. The formation of Ti vacancies takes place only to a very limited extent^9^ and Sr excess in STO thin films is therefore much more likely accommodated by the formation of extended Sr_*n*+1_Ti_*n*_O_3*n*+1_ Ruddlesden-Popper (RP) phases^10^,^11^ which comprise alternating TiO2 and SrO layers with an additional rock-salt layer every n^th^ SrO layer. Moreover, crystallographic shear plane defects resulting from coherent intergrowth of additional SrO layers have been observed in the literature^12^,^13^,^14^.

Interestingly, RP-type defects in STO have strong impact on the optical bandgap^15^, improve the figure of merit of thermoelectrics^3^ as well as the performance^16^ and reliability of memristive devices^17^. It is important to note that the impact of RP-type planar faults on the performance of thin film devices differs strongly between planar faults oriented parallel and perpendicular to the growth direction. For example, films with vertical SrO intergrowth defects significantly improve the switching ability of memristive devices compared to devices with thin films comprising only SrO planes aligned parallel to the substrate surface^16^. Furthermore, the thermal conductivity of Sr rich STO shows a striking decrease if the planar RP-defects are oriented perpendicular to the direction of heat flow^3^. Therefore, the possibility to tune the orientation of RP-type planar faults is of considerable interest for a broad variety of applications. It has been shown recently that growth temperature and oxygen pressure influence the preferential orientation of planar stacking faults in MBE grown thin films^3^, however, a microscopic understanding of the formation mechanism for RP-type defects in Sr rich thin films is missing so far.

In this work, we elucidated the formation mechanism of RP-type defects during pulsed laser deposition of Sr rich SrTiO_3_ thin films by a combined analysis of *in-situ* reflection high electron energy diffraction (RHEED), atomic force microscopy (AFM), low energy electron diffraction (LEED) and high resolution scanning transmission electron microscopy. This knowledge provides a pathway for engineering functional planar faults in Sr rich STO thin films.

## Results

### Surface evolution in the early stage of growth

**Figure 1:**
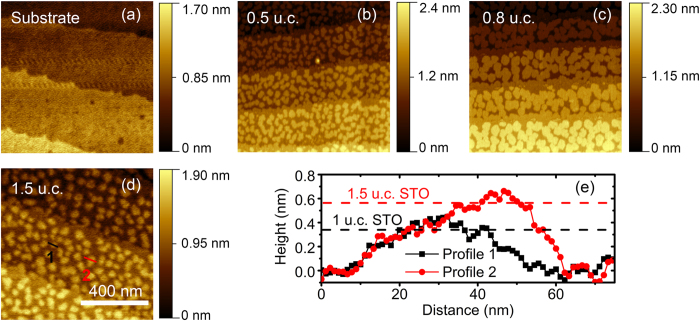
AFM analysis during growth of Sr rich STO. (**a**–**d**) AFM topographies for substrate, 0.5 u.c., 0.8 u.c. and 1.5 u.c. Sr rich STO on STO, respectively. (**a**,**d**) are measured by the *ex-situ* AFM, while (**b**,**d**) are measured by the *in-situ* AFM. (**e**) Line profiles for the markers in (**d**).

Sr rich SrTiO_3_ (STO) thin films are deposited by pulsed laser deposition (PLD) on TiO_2_ terminated STO single crystals with (001) surface orientation. The laser fluence during growth has been adjusted to 1 J/cm^2^, resulting in Sr rich films with 100% SrO termination determined by X-ray photoelectron spectroscopy (XPS)^18^. A detailed discussion of the thin film Sr/Ti stoichiometry will take place later within this manuscript. Figure 1 depicts the evolution of the surface morphology with increasing STO film thickness. Prior to the deposition, the TiO_2_ terminated substrate (Fig. 1(a)) exhibits flat terraces with 0.4 nm height, confirming a single termination situation. For the initial growth stage below one unit cell, namely 0.5 u.c. (Fig. 1(b)) and 0.8 u.c. (Fig. 1(c)), besides rarely occurring dirt particles (bright spots in Fig. 1(b)), solely islands with a height of 0.4 nm are observed on the terraces, corresponding to the height of the STO unit cell. Therefore, these islands should consist of completed STO unit cells. For the 1.5 u.c. thickness (Fig. 1(d)), small islands with 0.2 nm height are formed on the larger 0.4 nm islands, as can be seen in the red line profile in Fig. 1(e). The black line shows a line profile of a 0.4 nm island for comparison. These kind of half-unit cell islands with 0.2 nm height are absent for all sub-unit cell thin films in this study and in our previous investigations of stoichiometric STO thin films^19^. One possible explanation for the appearance of these 0.2 nm half-unit cell islands is the formation of SrO on the surface at the growth stage of 1.5 unit cells STO.

### Analysis of the surface reconstruction

**Figure 2:**
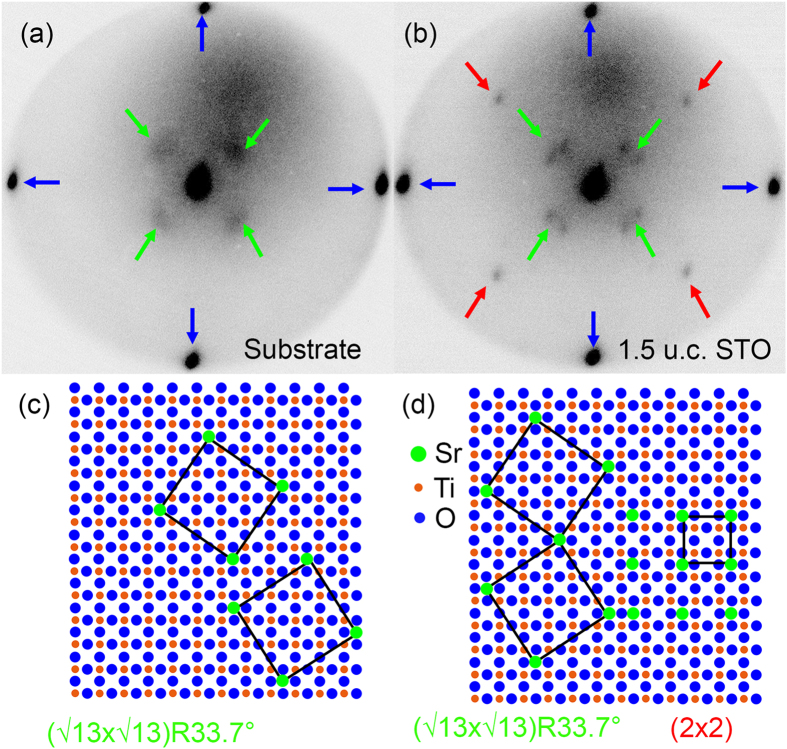
LEED analysis and illustration of the surface reconstruction. LEED pattern of (**a**) TiO_2_ terminated STO substrate and (**b**) 1.5 u.c. Sr rich STO thin film. The colors are reversed to intensify the contrast. The green arrows are for the ((√13 × √13)-R33.7°) and the red arrows are for the (2 × 2) reconstructions, respectively. The blue arrows are for (1 × 1) diffraction spots. (**c**,**d**) Model for possible surface reconstructions on substrate and Sr rich STO surfaces.

In order to clarify the origin of these additional half-unit cell islands, we performed low energy electron diffraction (LEED) measurements with a low-energy electron microscopy (LEEM) system on the substrate and the 1.5 u.c. STO thin film. The LEED pattern of the TiO_2_ terminated STO substrate and the 1.5 u.c. Sr rich STO thin film are shown in Fig. 2(a,b), respectively. For both, substrate surface and STO thin film surface, (1 × 1) diffraction pattern can be observed as four deep black spots marked with blue arrows in the LEED pattern in Fig. 2(a,b), respectively. Besides (1 × 1) spots, the substrate surface exhibits diffuse (√13 × √13)-R33.7° reconstruction (green arrows in Fig. 2(a)), whereas the Sr rich STO thin film surface exhibits well pronounced (√13 × √13)-R33.7° (green arrows in Fig. 2(b)) and (2 × 2) (red arrows in Fig. 2(b)) reconstructions. Since the (1 × 1) diffraction pattern is formed for both SrO and TiO_2_ termination^20^,^21^, the (1 × 1) pattern provides no information about the surface termination. On the contrary, the (√13 × √13)-R33.7° reconstruction supplies relevant information about the elemental surface composition. It has been shown by Kubo *et al*.^21^ that the increase of surface Sr atom concentration from 0.077 to 0.75 on the SrTiO_3_ surface results in a transition from (√13 × √13)-R33.7° to (2 × 2) reconstruction. Therefore, the appearance of the (2 × 2) reconstruction in Fig. 2(b) indicates an increase of the Sr atom concentration on the surface of the Sr rich STO thin film in comparison to the substrate.

Moreover, the four diffraction spots of the (√13 × √13)-R33.7° reconstruction in Fig. 2(a) are diffuse without splitting, whereas the (√13 × √13)-R33.7° reconstruction in Fig. 2(b) shows splitting of each spot. The splitting of the (√13 × √13)-R33.7° diffraction spots results from well-ordered two-fold domains of this reconstruction (Fig. 2(d), two larger squares on the left). The diffuse nature of the diffraction spots in Fig. 2(a) therefore suggests a long distance between different (√13 × √13)-R33.7° domains inhibiting a pronounced long range order reconstruction. Thus the concentration of Sr atoms that form the (√13 × √13)-R33.7° reconstruction on the 1.5 u.c. Sr rich STO thin film is strongly increased with respect to the substrate surface.

As a short summary, the appearance of the (2 × 2) reconstruction on Sr rich STO thin film and the diffuse nature of the (√13 × √13)-R33.7° reconstruction indicates a strongly increased Sr concentration on the surface of the 1.5 u.c. Sr rich STO film in comparison to the substrate. Considering the high oxygen partial pressure (10^−1^ mbar) during growth, it can be assumed that an increased concentration of Sr atoms on the surface goes along with the formation of SrO islands. Therefore the 0.2 nm islands in Fig. 1(e) can be identified as SrO islands.

We thereby conclude that SrO island have formed on the surface of the 1.5 u.c. thin film, whereas they can not be detected for film thicknesses in the sub-monolayer regime. In the sub-u.c. regime, the Sr excess becomes apparent by a reduced surface diffusion coefficient and a delayed island coalescence, hinting at the formation of Ti vacancies^19^. In our earlier work, we could explicitly show that Ti vacancies are generally present in our PLD grown STO thin films and become the dominant defect type for slightly Sr rich thin films^5^,^9^. We can therefore not exclude that a certain amount of Ti vacancies is present even at larger STO thicknesses. However, according to the high formation energy of Ti vacancies^22^ and the resulting lattice strain on the one hand and the low formation energy of SrO on the surface of STO [19]^23^ on the other hand, an excess of Sr should result in a surface segregation rather than in the formation of Ti vacancies in thermodynamic equilibrium as observed on (110) oriented donor doped STO thin film surfaces^24^. One possible explanation for our experimental findings is that the strain induced by the formation of Ti vacancies is negligible in the sub-u.c. cell regime and that Sr segregation starts when a complete unit cell is formed. Another possible explanation is that SrO islands are already present in the sub-u.c. regime, but that the island size is below the resolution limit of the AFM of around 10 nm. With increasing STO coverage, the total amount of Sr excess increases, resulting in the formation of larger SrO islands, which then can be detected by AFM. This progressive SrO surface segregation is also consistent with the observation of a change of the surface termination from TiO_2_ to SrO for slightly Sr rich 20 nm thick thin films^18^.

### Accomodation of Sr excess during progressive thin film growth

**Figure 3:**
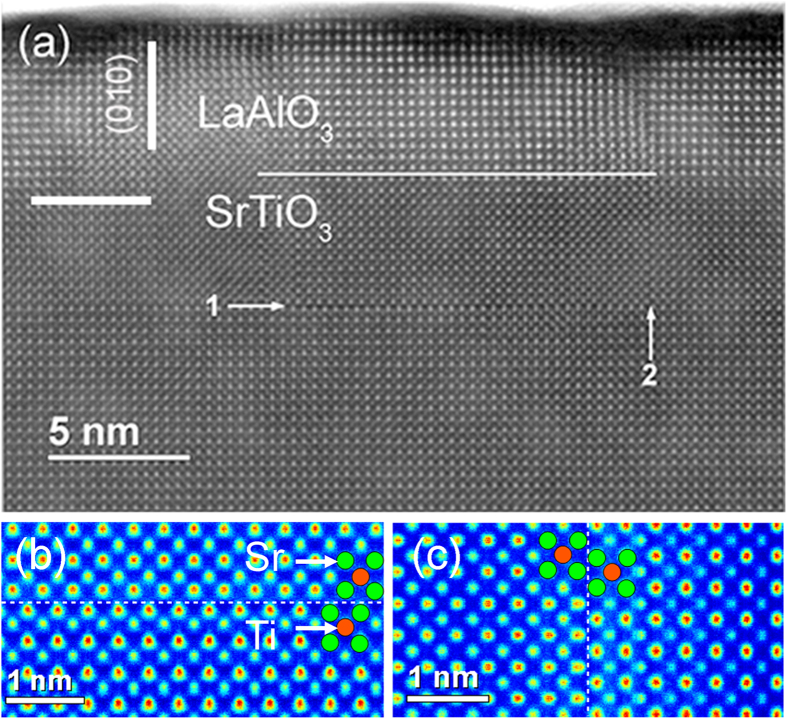
Microstructure of Sr rich thin films determined by HRSTEM. (**a**) HAADF image of part of a 50 u.c. Sr rich STO (001) thin film with 10 u.c. of LAO on top obtained by STEM. We see two kinds of antiphase boundaries APB 1 and APB 2. (**b**,**c**) are HAADF images averaged along the APB 1 and APB 2, respectively (images are encoded in Jet color scale for easy recognition of the type of atomic columns (green: Sr, orange: Ti-O)).

**Figure 4:**
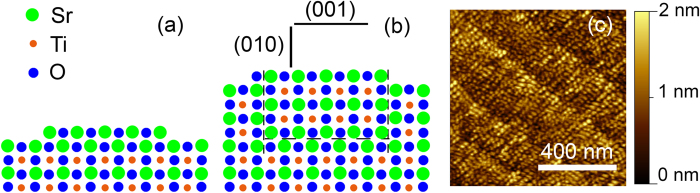
APB formation mechanism. (**a**) Sketch for the atomic configuration of the surface rock-salt-type SrO double layer island surrounded by a SrO single layer, which is the prerequisite for the formation of APBs. (**b**) Sketch for the atomic configuration resulting in the formation of APB1 in (001) and APB2 in (010) during growth. (**c**) Surface morphology of the 20 nm Sr rich STO thin film determined by *in-situ* AFM shown in Fig. 3 prior to the LAO deposition.

In order to investigate the impact of SrO segregation on the defect formation during progressive growth, we performed STEM measurements on a Sr-rich STO thin film covered with a LaAlO_3_ (LAO) protection layer. Figure 3(a) shows the high-angle annular dark field (HAADF) image of a Sr rich STO thin film with a 10 u.c. LAO protection layer. HAADF images depend on the composition through Z^ζ^ of the scattering cross section, where Z is the atomic number and ζ is close to 2 depending on the actual value of the collection angle of the HAADF detector. As a result, the intensity of atomic columns follows the order La > Sr > Ti-O > Al-O. The boundary between LAO and STO is qualitatively marked with the horizontal white line, keeping in mind that the surface of both STO and LAO is not flat and with a peak-to-valley roughness of about 2 nm as can be estimated by the surface on the STO thin film prior to LAO deposition (see Fig. 4(c)).

The following discussion is focused on the structural details of the Sr rich STO below the LAO protection layer. We see two different orientations of the anti-phase boundaries (APB), which are on the (001) plane (APB 1) and the (010) plane (APB 2), respectively. The HAADF images, averaged along APB 1 and APB 2, are presented in Fig. 3(b,c), respectively. The orange circles represent the Ti-O column and the green circles represent the Sr column in [100] direction. We see in Fig. 3(b) on the APB1 that two Sr rows are accommodated with a 0.5 u.c. parallel shift in [010] direction (a/2[010]). On the APB2 (Fig. 3(c)), two Sr columns are arranged with a 0.5 u.c. parallel shift in [001] direction (a/2[001]). Considering the ABPs being Ruddlesden-Popper type, the lattices will have a/2[111] displacement relative to each other across the APBs.

The formation of these two types of APBs during thin film growth can be explained by considering that a rock-salt-type SrO layer is rather formed on the surface of single islands than on the whole STO surface. This is consistent with the formation of single layer SrO islands observed in the early growth stage (Fig. 1(c,d)) and the morphology of the 20 nm thick STO film shown in Fig. 4(c). This scenario is sketched in Fig. 4(a,b). As a result of the rock-salt-type SrO island in the (001) plane, which has an a/2[111] lattice shift between the two single SrO layers, the APB1 with a/2[010] as lattice shift and the APB2 with a/2[001] as lattice shift are formed in the STO. The a/2[100] lattice shift parallel to the electron beam is not resolvable in the projection.

Figure 3(a) shows that the lateral extension of the APB 1 in the (001) plane is around 10 nm. However, only one clear APB 2 between the central region and the right region is visible in Fig. 3(a), whereas on the left side of the APB1 a diffuse region with overlapped intensities of Sr and Ti-O columns is observed. This indicates that the cross section for TEM meets only the APB 2 on the right side with perpendicular configuration. On the left end of APB 1, the other APB 2 is not edge-on showing an overlap of the two lattices across the boundary because of the faceting of the boundary along the projection axis. Additional image analysis to corroborate this conclusion can be found in Fig. S1 within the supplementary information.

It is important to note that both orientations of APBs in the (001) plane (APB 1) and in the (010) plane (APB 2) are observed only within a region of 12 u.c. beneath the surface of the 50 u.c. thick film. As can be clearly seen in Fig. 3(a), below the upper 12 u.c., the STO thin film is completely homogeneous and has a high lattice perfection. This observation is consistent with previous reports about the inset of SrO planar faults in Sr rich STO thin films above a thickness of around 15 u.c.^25^ and our previous observation, that Sr rich thin films exhibited a different contrast in TEM after a thickness of about 30 u.c.^26^. Although the starting thickness might vary with the non-stoichiometry and the growth conditions, this seems to be a generic effect taking place in Sr rich STO thin films.

As a result of the vertical Sr concentration gradient observed by TEM, one has to be careful with the interpretation of the stoichiometry determined by surface sensitive techniques such as X-ray photoelectron spectroscopy (XPS). Although we have developed a method to separate between STO thin film stoichiometry and the termination layer by employing angle-dependent XPS measurements^18^, the information depth is in the order of 6 u.c. Based on this method, we have concluded that the STO thin films presented in this study are SrO terminated and have an Sr excess of 17%. However, assuming that the whole Sr excess in the STO thin film is confined in the upper 12 u.c., the overall Sr/Ti concentration is strongly overestimated by the surface sensitive XPS analysis. In order to estimate the average Sr surplus in the films provided by the laser plume, the most simple approximation is to consider a bilayer of a 38 u.c. perfect stoichiometric STO and a 12 u.c. layer with 17% Sr excess. As a result of this simplified approximation, the average Sr excess can be estimated to 4%. However, this value has to be regarded as a rough estimation, since the presence of Ti vacancies in the first 38 u.c. can not be excluded according to the insufficient sensitivity of STEM to low concentrations of point defect vacancies.

## Discussion

**Figure 5:**
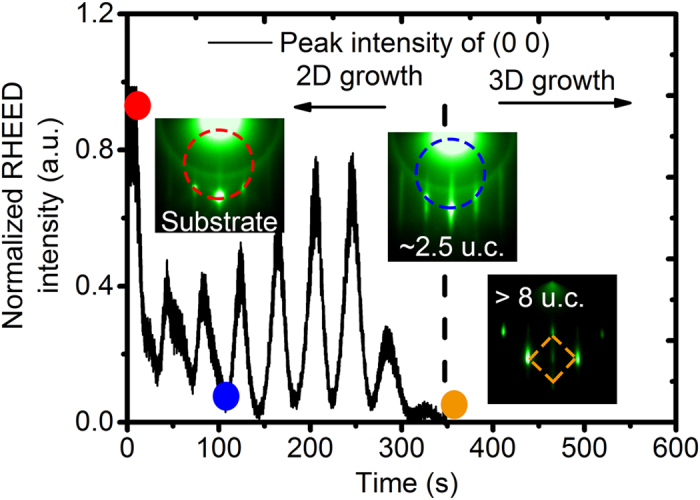
RHEED intensity oscillations for a Sr rich STO thin film. The corresponding RHEED patterns are shown in the insets. The balls with red, blue and orange colors on the RHEED oscillation curve indicate the position for the RHEED pattern of substrate, 2.5 u.c. STO and ~500 u.c. STO, respectively.

In order to clarify a possible correlation between PLD growth mode and the onset of APB formation we consider the reflection high energy electron diffraction (RHEED) analysis performed during PLD growth of Sr rich STO thin films depicted in Fig. 5. The RHEED pattern at the early growth stage shows diffraction spots on the Laue rings (~ 2.5 u.c., middle inset). For this specific thickness, we see also two weak streaky spots between the most bright diffraction spots of (1 0), (0 0) and (−1 0) on the Laue ring, which hint on the presence of a surface reconstruction. However, these streaky spots are not sufficient for an identification of the surface reconstruction. The peak intensity of the specular spot oscillates till ~9 u.c. and then decreases to zero. A four-fold symmetric diffraction pattern appears after ~8 u.c., which indicates the formation of a 3D cubic structures and a change in the growth mode from 2D layer growth to 3D island growth at ~8 u.c. Thus the thickness of growth mode transition is not the same as the start of APB formation and even below the expected surface termination change from TiO_2_ to SrO at about 25 u.c. for the above estimated mean value of Sr/Ti~1.04, assuming a complete flow of Sr to the surface after 1.5 u.c.

Therefore, we conclude that the grow mode transition, which is not observed for stoichiometric thin films^19^, takes place as soon as the formation of SrO islands on the STO surface, observed by AFM and LEED, becomes dominant after 1.5 u.c. The presence of SrO islands might inhibit the lateral adatom diffusion, as it has been observed for the growth of SrRuO_3_ thin films^27^, thereby inducing a transition to 3D island growth. Since the formation of APBs requires the formation of SrO double layer islands with rock-salt structure (Fig. 4(a)), a much higher Sr concentration in the surface near region is needed for the APB formation than for the growth mode transition. Since the Sr concentration at the surface should increase with increasing STO thickness, the formation of SrO rock-salt layer islands should take place at even higher film thicknesses. In particular, it has been shown in the literature that due to the high formation energy of rock-salt layers, even a trilayer of SrO is needed to prevent a layer exchange between SrO and TiO_2_ during the subsequent deposition of TiO_2_ by molecular beam epitaxy^23^,^28^. Assuming that the whole excess of Sr would flow to the surface during STO growth, the STO thickness has to exceed 75 u.c. in order to form a homogeneous SrO trilayer. Thus, it is reasonable that islands with stable rock-salt structure are formed at a thickness of about 38 u.c. We can therefore describe the defect formation process during the growth of Sr rich STO by the following two stages:

- From 1.5 u.c. to ~ 38 u.c. the surface is progressively enriched with Sr, resulting in the formation of single layer SrO islands on top of STO islands and a well pronounced (2 × 2) surface reconstruction. This is the main route to accommodate Sr excess in STO at this growth stage. The formation of the SrO islands results in a transition from 2D growth mode to 3D growth mode after 8 u.c. as a result of the inhibited adatom diffusion.
- At around 38 u.c., the Sr excess on the surface is sufficient to form islands with stable SrO double layer rock-salt structure on the STO surface (Fig. 4(a)). The STO layers grown on the rock-salt-type double layer SrO islands (marked region with broken lines in Fig. 4(b)) and the neighboring undisturbed STO lattice experience a phase shift between SrO and TiO_2_ sub-monolayers. As a result, two types of APBs, namely APB1 in (001) plane as well as APB2 in (010) plane, are formed and accommodate the Sr excess in the upper 12 u.c. of the STO thin film.

Based on our suggested microscopic model, it can be expected that the film thickness at which the formation of ABPs starts will decrease with increasing Sr excess. We can furthermore conclude that the formation of (010) APBs is a result of the presence of SrO double layer islands with rock-salt structure and that the size and the density of these islands will determine the density of the (010) APBs. Since the size and the density of the islands will be influenced by the growth kinetics^29^ it is expected that the APB1 density induced during PLD growth will strongly depend on the substrate temperature, but also on the plume kinetics influenced by oxygen pressure pressure and laser fluence. It is interesting to note that the density of (010) APBs indeed decreases with increasing growth temperature during MBE growth^3^ which is consistent with our microscopic model for their formation process. This type of defect is therefore inherently connected with the kinetically limited thin film growth and not observed in thermodynamic equilibrium in Sr rich STO^30^,^31^,^32^. Therefore, our findings about the APB formation mechanism related to kinetically limited growth of SrO rock-salt islands pave the way towards engineering of RP type defects in Sr rich STO thin films by modifying the growth kinetics.

## Methods

STO thin films with various thicknesses are deposited by PLD on STO single crystals with (001) surface orientation (*Crystec GmbH*). The same crystals are used as target in the PLD system equipped with a Kr-F excimer laser of 248 nm wavelength (*CompexPro205F COHERENT*^*®*^). During the PLD process, the oxygen pressure is 0.1 mbar with a UHV of 10^−8^ mbar background pressure. The substrate-to-target distance is fixed to 44 mm. The laser repetition rate is 1 Hz. The laser fluence used for the ablation is set to be 1 J/cm^2^. To provide a TiO_2_ termination of the STO substrate, etching with buffered HF and subsequent annealing for 2 h in air is carried out. During the deposition process, the substrates are heated from the back side to 800 °C with a laser heater (140 W, 925 nm). After deposition, the power of the laser heater is turned off, resulting in a cooling rate of ~10 K/s guaranteeing the preservation of the surface morphology to a large extent. Based on our previous investigation, this set of parameters results in STO thin films with 100% SrO termination and a Sr/Ti ratio of 1.17^18^. For the subsequent deposition of a LAO protection layer employed for the STEM analysis, a LAO single crystal is used as target. The repetition rate is 1 Hz and the laser fluence is 1.9 J/cm^2^. The deposition of 10 u.c. LAO is carried out at 700 °C and 10^−4^ mbar *p*O_2_. AFM analysis was performed either *ex-situ* on a *S.I.S* system in non-contact mode with a Si tip (radius < 10 nm) in air (f = 164 kHz, F = 9 nN) or *in-situ* on an *Omicron* system in contact mode with a single crystalline diamond tip (*NaDiaProbes*^*®*^ from *nanoScience instruments*^*©*^; radius < 10 nm).

Cross-sectional TEM specimens were prepared by focused ion beam (FIB) milling using an FEI Helios NanoLab 400 S system with a Ga ion beam^33^. TEM specimens were further thinned and cleaned with an Ar ion beam in a Fischione Nanomill 1040 at 900 eV and 500 eV beam energies, respectively. STEM imaging was conducted with the FEI Titan G3 50–300 PICO microscope^34^ operated at 200 kV accelerating voltage.

The LEEM experiments were performed in the ‘Escher’ setup, an aberration-corrected LEEM facility at Leiden University^35^. Samples were heated to 500 °C in a oxygen background pressure of 5 × 10^−5^ mbar to remove any surface contaminants and prevent the sample from charging. Diffraction images where taken at 12 eV.

## Additional Information

**How to cite this article**: Xu, C. *et al*. Formation mechanism of Ruddlesden-Popper-type antiphase boundaries during the kinetically limited growth of Sr rich SrTiO_3_ thin films. *Sci. Rep.* **6**, 38296; doi: 10.1038/srep38296 (2016).

**Publisher's note:** Springer Nature remains neutral with regard to jurisdictional claims in published maps and institutional affiliations.

## Supplementary Material

Supplementary Information
