# Supplementary material for: Formation mechanism of Ruddlesden-Popper-type antiphase boundaries during the kinetically limited growth of Sr rich SrTiO3 thin films
Source: Sci Rep. 2016 Dec 6;6:38296. doi: 10.1038/srep38296 (PMC5138825; doi:10.1038/srep38296)
Supplement: Supplementary Information [file srep38296-s1.pdf]

Formation mechanism of Ruddlesden-Popper-type antiphase boundaries during the kinetically limited growth of Sr rich $SrTiO_3$ thin films

Chencheng Xu[a], *Hongchu Du*[b,c], *Alexander. J. H. van der Torren*[d], *Jan Aarts*[d], *Chun-Lin Jia*[b,e], *Regina Dittmann**[a]

[a]Peter Grünberg Institute (PGI-7), Forschungszentrum Jülich GmbH, 52425 Jülich, Germany
[b]Ernst Ruska-Centre (ER-C) for Microscopy and Spectroscopy with Electrons, Forschungszentrum Jülich GmbH, 52425 Jülich, Germany
[c]Central Facility for Electron Microscopy (GFE), RWTH Aachen University, 52074 Aachen, Germany
[d]Leiden University, Huygens Kamerlingh Onnes Lab, NL-2300 RA Leiden, Netherlands
[e]Peter Grünberg Institut (PGI-5), Forschungszentrum Jülich GmbH, D-52425 Jülich, Germany
*Correspondence and requests for materials should be addressed to R.D. (email: r.dittmann@fz-juelich.de)

Supplementary Information:

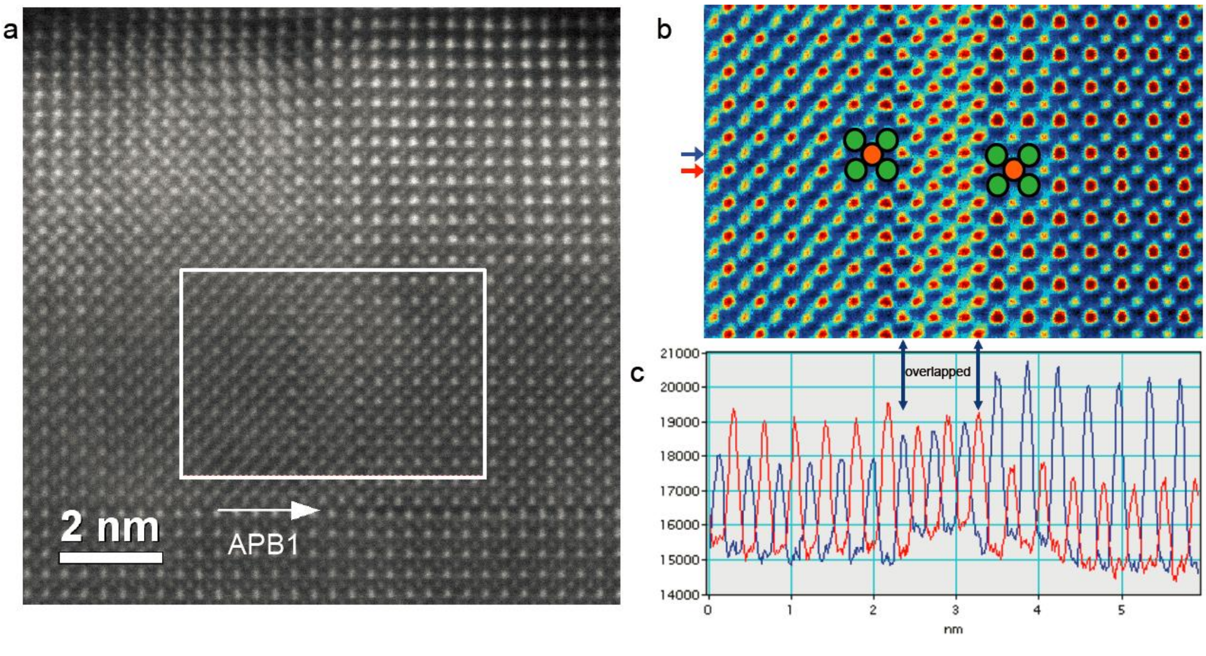

Fig. S1 The other APB 2 on the left end of APB 1 in Fig. 3 is not edge-on showing an overlap of the two lattices across the boundary because of the faceting of the boundary along the projection axis. (a) HAADF-STEM image of the APB2 (in the marked frame) on the left end of APB1. (b) HAADF image averaged along the APB 2 encoded in Jet color scale for easy recognition of the type of atomic columns (green: Sr, orange: Ti-O)). (c) Line-Profiles along the atomic planes indicated by respective arrows of the same color in (b). The width of the overlapped region is of 2.5 u.c. As a result of the overlap of the two lattices, the intensity of the atomic columns become similar each other with values between that of the normal Ti-O and Sr columns. Keep in mind that the lattices have an a/2[111] across the APB.The displacement of a/2[100] in the projection direction parallel the electron beam is not resolvable.
